# EORTC PET response criteria are more influenced by reconstruction inconsistencies than PERCIST but both benefit from the EARL harmonization program

**DOI:** 10.1186/s40658-017-0185-4

**Published:** 2017-05-30

**Authors:** Charline Lasnon, Elske Quak, Pierre-Yves Le Roux, Philippe Robin, Michael S. Hofman, David Bourhis, Jason Callahan, David S. Binns, Cédric Desmonts, Pierre-Yves Salaun, Rodney J. Hicks, Nicolas Aide

**Affiliations:** 1 Nuclear Medicine Department, François Baclesse Cancer Centre, Caen, France; 2 INSERM U1086 ANTICIPE, BioTICLA, Caen University, Caen, France; 3 Nuclear Medicine Department and EA 3878 IFR 148, University Hospital, Brest, France; 4 Cancer Imaging, Peter Mac Callum Cancer Institute, Parkville, Australia; 5 Nuclear Medicine Department, University Hospital, Caen, France; 6 The Sir Peter MacCallum Department of Oncology, the University of Melbourne, Melbourne, Australia; 7 Normandy University, Caen, France; 8 Nuclear Medicine Department, Caen University Hospital, Avenue Côte de Nacre, 14000 Caen, France

**Keywords:** PET, ^18^F-FDG, Therapy response, PERCIST, EORTC, Harmonization

## Abstract

**Background:**

This study evaluates the consistency of PET evaluation response criteria in solid tumours (PERCIST) and European Organisation for Research and Treatment of Cancer (EORTC) classification across different reconstruction algorithms and whether aligning standardized uptake values (SUVs) to the European Association of Nuclear Medicine acquisition (EANM)/EARL standards provides more consistent response classification.

**Materials and methods:**

Baseline (_PET1_) and response assessment (_PET2_) scans in 61 patients with non-small cell lung cancer were acquired in protocols compliant with the EANM guidelines and were reconstructed with point-spread function (PSF) or PSF + time-of-flight (TOF) reconstruction for optimal tumour detection and with a standardized ordered subset expectation maximization (OSEM) reconstruction known to fulfil EANM harmonizing standards. Patients were recruited in three centres. Following reconstruction, EQ.PET, a proprietary software solution was applied to the PSF ± TOF data (PSF ± TOF.EQ) to harmonize SUVs to the EANM standards. The impact of differing reconstructions on PERCIST and EORTC classification was evaluated using standardized uptake values corrected for lean body mass (SUL).

**Results:**

Using OSEM_PET1_/OSEM_PET2_ (standard scenario), responders displayed a reduction of −57.5% ± 23.4 and −63.9% ± 22.4 for SUL_max_ and SUL_peak_, respectively, while progressing tumours had an increase of +63.4% ± 26.5 and +60.7% ± 19.6 for SUL_max_ and SUL_peak_ respectively. The use of PSF ± TOF reconstruction impacted the classification of tumour response. For example, taking the OSEM_PET1_/PSF ± TOF_PET2_ scenario reduced the apparent reduction in SUL in responding tumours (−39.7% ± 31.3 and −55.5% ± 26.3 for SUL_max_ and SUL_peak_, respectively) but increased the apparent increase in SUL in progressing tumours (+130.0% ± 50.7 and +91.1% ± 39.6 for SUL_max_ and SUL_peak_, respectively).

Consequently, variation in reconstruction methodology (PSF ± TOF_PET1_/OSEM_PET2_ or OSEM _PET1_/PSF ± TOF_PET2_) led, respectively, to 11/61 (18.0%) and 10/61 (16.4%) PERCIST classification discordances and to 17/61 (28.9%) and 19/61 (31.1%) EORTC classification discordances. An agreement was better for these scenarios with application of the propriety filter, with kappa values of 1.00 and 0.95 compared to 0.75 and 0.77 for PERCIST and kappa values of 0.93 and 0.95 compared to 0.61 and 0.55 for EORTC, respectively.

**Conclusion:**

PERCIST classification is less sensitive to reconstruction algorithm-dependent variability than EORTC classification but harmonizing SULs within the EARL program is equally effective with either.

**Electronic supplementary material:**

The online version of this article (doi:10.1186/s40658-017-0185-4) contains supplementary material, which is available to authorized users.

## Background

^18^F-FDG PET is increasingly being used for response evaluation in cancer patients, in clinical routine or in clinical trials [1–6]. Two main schemas based on the degree of standardized uptake value (SUV) change following treatment are currently used: the European Organisation for Research and Treatment of Cancer (EORTC) criteria [7] and PET evaluation response criteria in solid tumours (PERCIST) [8]. However, many sources of error in SUV measurement exist [9–11]. In particular, technological improvements can lead to significant device-dependent and reconstruction-dependent variations in quantitative values [12–14]. This could lead to classification errors by exceeding thresholds used for discriminating between responding and non-responding tumours unless acquisition and processing of pre- and post-treatment scans are acquired on the same scanner and processed identically.

The European Association Research Ltd (EARL) accreditation program [15] is an SUV harmonization strategy aiming at minimizing the variability in SUV measurements by harmonizing patient preparation and scan acquisition and processing [16]. While many sources of error in SUV measurements are overcome by complying with the EANM guidelines for PET tumour imaging [17–19], reconstruction-dependent variations require either the use of an additional filtering step [20] or the generation of two sets of images: one to provide optimal diagnostic quality and another to meet quantitative harmonization standards [21]. Previous research from the collaborators in this study have shown that SUV_max_ is more sensitive to reconstruction inconsistency than SUV_peak_ [20] and that reconstruction inconsistencies may affect PERCIST classification [22]. Consequently, one could expect a more significant impact of these inconsistencies on EORTC classification, which is based on SUV_max_ variation, than on PERCIST, which is based on SUV_peak_.

The aim of this study was to evaluate the impact of SUV reconstruction dependency on PERCIST and EORTC classification and the ability of the EARL program to minimize variability in response assessment. To assess this, we reconstructed the same PET raw data with an OSEM algorithm known to meet EANM requirements and also with PSF with or without TOF reconstruction (PSF ± TOF). Post-reconstruction filtering was then applied to the PSF ± TOF reconstruction with EQ.PET (Siemens Medical Solutions), a proprietary software solution allowing visualization of optimized images while simultaneously obtaining harmonized SUV values [20, 23].

## Methods

### Patients

Sixty-one patients with non-small cell lung cancer (NSCLC) who were scanned for monitoring efficacy of chemotherapy, molecularly targeted therapies or radiotherapy were included. The cohort was comprised of 51 patients prospectively included in a multicentre study involving three PET centres and 10 patients included in a single-centre prospective study. Informed consent was waived for this type of study by the local ethics committee (Ref A12-D24-VOL13, *Comité de protection des personnes Nord-Ouest III*) since the scans were performed for clinical indications, and the study procedures were performed independently without influencing clinical reporting.

Patient’s sex ratio (male/female) was 2.4:1; mean ± SD age was 62.7 ± 9.4 years. The interval between the pre- and post-treatment PET scans was 103 ± 53 days. Fifty-eight (95.1%) patients underwent chemotherapy, 1 (1.6%) patient had radiotherapy and 2 (3.3%) patients were administered targeted therapies (TKI and immunotherapy).

### PET systems

Data from the following three PET systems were used for this study: a Biograph 6 TrueV with PSF reconstruction, a mCT with PSF + TOF, and a Biograph 64 TrueV with PSF reconstruction (Siemens Medical Solutions). Both the Biograph systems were equipped with an extended axial field-of-view.

### Patient preparation, PET acquisition and reconstruction parameters

All patients were requested to fast for 6 h prior to the ^18^F-FDG injection. Patient height, weight and blood glucose levels were recorded. Patients were injected intravenously with ^18^F-FDG, followed by a 60 min rest in a warm room.

A daily calibration of each PET system was performed with a ^68^Ge source according to the manufacturer’s protocol. A quarterly cross-calibration of each PET system was performed according to the EANM guidelines, as described elsewhere [17, 18], and clocks from workstations were synchronized weekly.

Patients were scanned from the skull vertex or base to the mid-thighs. All raw PET data were reconstructed with the local PSF ± TOF settings for optimal lesion detection and an OSEM-3D reconstruction algorithm fulfilling the EANM guidelines regarding recovery coefficients (Table 1). Scatter and attenuation corrections were applied on all PET acquisitions.

**Table 1:** PET/CT acquisition and reconstruction parameters for the three participating centres

Site and system		Centre 1 biograph 6		Centre 2 biograph mCT		Centre 3 biograph 6			
PET acquisition	Duration per bed position	2 min and 40 s (BMI ≤25) or 3 min and 40 s (BMI >25)		2 min and 00 s		2 min and 30 s (≤65 kg), 3 min (65–85 kg), 3 min and 30 s (85–100 kg), 4 min and 00 s (>100 kg)			
PET reconstruction	Details	–	–	–	–		≤65 kg	65–100 kg	>100 kg
	Reconstruction	OSEM3D	PSF	OSEM3D	PSF + TOF	OSEM3D	PSF	PSF	PSF
	Iterations/subsets	4i 8s	3i 21s	2i 24s	2i 21s	4i 8s	3i 21s	3i 21s	3i 21s
	Post-filter	5 mm	0 mm	4.4 mm	2 mm	3.5 mm	6 mm	5 mm	4 mm
	Matrix	168 × 168	168 × 168	200 × 200	200 × 200	168 × 168	168 × 168	168 × 168	168 × 168
	Pixel spacing	4.07 × 4.07	4.07 × 4.07	4.07 × 4.07	4.07 × 4.07	3.39 × 3.39	3.39 × 3.39	3.39 × 3.39	3.39 × 3.39
	Slice thickness	5 mm	5 mm	2.027 mm	2.027 mm	3 mm	3 mm	3 mm	3 mm
	EQ filter	0 mm	6.9 mm	0 mm	6.3 mm	0 mm	2.4 mm	3.9 mm	4.9 mm

### EQ.PET methodology

For each PET system, the EQ.PET filter was calculated on the phantom data of each PSF ± TOF reconstruction as described in details elsewhere [21]. Briefly, the recovery coefficients (RCs; defined as the ratio between the measured and true activity concentration for each sphere) of a National Electrical Manufacturers Association NU2 phantom scanned as per EANM guidelines were aligned to the EANM reference RCs by applying a Gaussian filter.

### PERCIST and EORTC evaluation

All PET exams were analyzed on Syngo.via software equipped with EQ.PET (Siemens Medical Solutions). For interpretation purposes, both the reconstruction for optimal lesion detection (PSF ± TOF) and the OSEM reconstruction were displayed on the screen together with the EQ.PET-filtered harmonized SUV results for the tumour region(s) of interest. The EQ.PET-filtered images were not displayed on the screen.

For PERCIST criteria [8], the measurable target lesion is the single most intense tumour site on pre- and post-treatment scans, which means that the target lesion is not necessarily the same pre- and post-treatment. As per EORTC PET response criteria, the volumes of interest (VOI) should involve the same tumour lesion on pre- and post-treatment scan.

In practice, the target lesion on baseline scan was chosen as the most intense lesion and located by scaling the 3D MIP view both on the OSEM and PSF ± TOF reconstructions. VOIs were drawn on one reconstruction and automatically propagated to the second set of reconstruction (propagation from OSEM to PSF ± TOF and vice versa). Within these volumes of interest, lean body mass SUV_peak_ (SUL_peak_) and SUL_max_ were measured.

The same VOI methodology was used on the post-treatment scan, where the target lesion was chosen as the most intense lesion for PERCIST, while the same target lesion for baseline and post-treatment scans was used for EORTC classification.

Based on the SUL_peak_ and SUL_max_ variation between the pre- and post-treatment scans, patients were classified according to PERCIST and EORTC as follows:

- *Complete metabolic response* (CMR): complete resolution of ^18^F-FDG uptake in the tumour volume, with tumour SUL lower than liver SUL and background blood pool, and disappearance of all lesions if multiple.
- *Partial metabolic response* (PMR): at least 30% (PERCIST) or 25% (EORTC) reduction in tumour uptake.
- *Stable metabolic disease* (SMD): less than 30% (PERCIST) or 25% (EORTC) increase, or less than 30 or 25% (EORTC) decrease in tumour 18F-FDG SUL_peak_ and no new lesions.
- *Progressive metabolic disease* (PMD): greater than 30% (PERCIST) or 25% (EORTC) increase in ^18^F-FDG tumour SUL_peak_ within the tumour or appearance of new lesions.

### Statistical analysis

Quantitative data from clinical PET/CT examinations are presented as mean (standard deviation ± SD). The relationship between PSF ± TOF, PSF ± TOF.EQ and OSEM quantitative values were assessed with Bland-Altman plots. Levels of agreement between the different types of reconstruction were evaluated using the kappa statistic. The use of OSEM reconstruction for both pre- and post-therapeutic PET examinations (OSEM_PET1_/OSEM_PET2_) was used as the “current standard” to classify the therapeutic response of each lesion and compared to other scenarios. Kappa values were reported using the benchmarks of Landis and Koch [24].

Graphs and analyses were carried out using Prism GraphPad and the Vassar University website for statistical computation (http://vassarstats.net).

## Results

### Ability of the EQ.PET methodology to harmonize SUL assessments

**Fig. 1:**
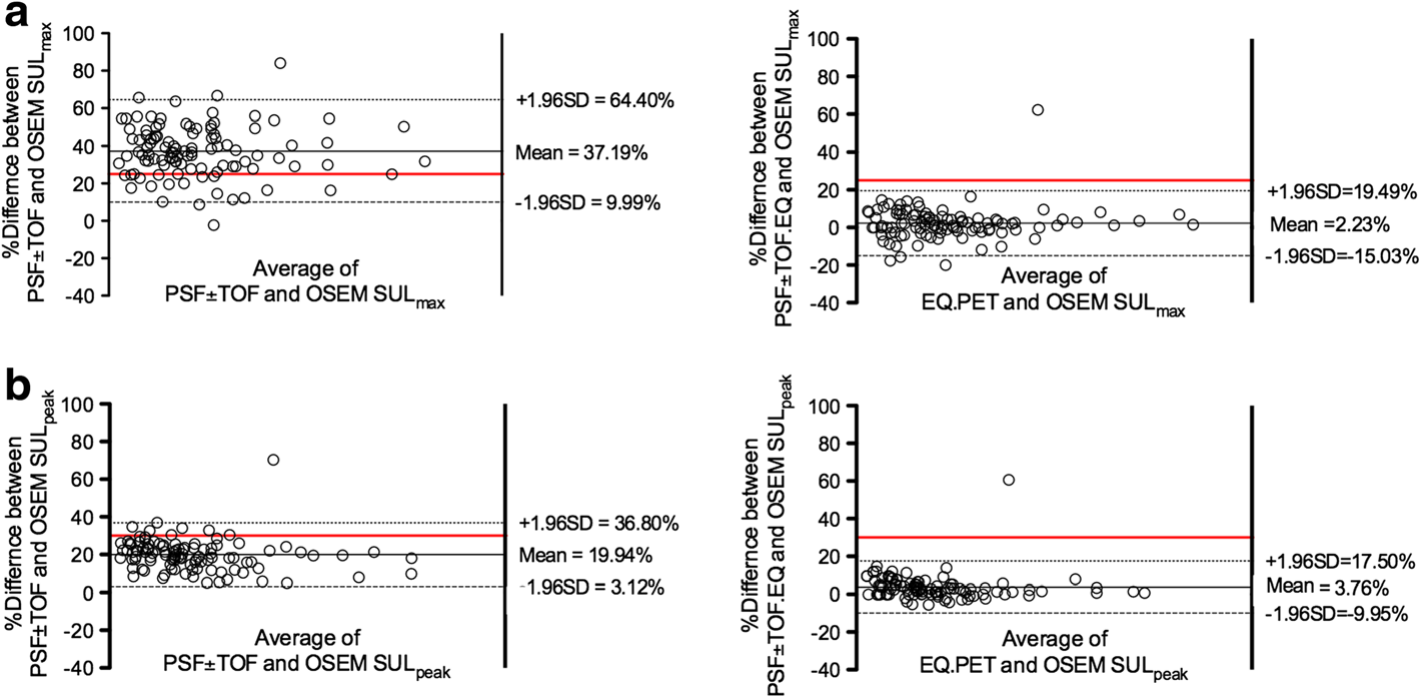
Relationship between SUL_max_ and SUL_peak_ in lesions extracted from PSF ± TOF or PSF ± TOF.EQ and OSEM images, assessed using Bland-Altman plots. Mean percentage difference between SUL_max_ (**a**) and SUL_peak_ (**b**) obtained with a conventional OSEM algorithm and those obtained with PSF ± TOF reconstructions are shown before and after application of the EQ.PET methodology. The red lines denote the 25% and 30% thresholds used to discriminate between stable metabolic disease and progressive metabolic disease with EORTC classification and PERCIST, respectively

The mean percentage difference (% difference) between PSF ± TOF and OSEM reconstructions were 37.19% (95%CI 9.99–64.40) and 19.94% (95%CI 3.12–36.80) for SUL_max_ and SUL_peak_, respectively. After application of the EQ.PET filter, this was reduced to 2.23% (95%CI −15.03–19.49) and 3.76% (95%CI −9.95–17.50) for SUL_max_ and SUL_peak_, respectively (Fig. 1). Noticeably, in both cases, confidence intervals were slightly narrower for SUL_peak_ values.

### Impact of reconstruction-dependent variation on SUL changes between baseline and post-treatment scans

The same target lesion for baseline and post-treatment scans was used for EORTC classification except for two patients. The first patient displayed a large tumoural and nodal complex for which the EQ.PET software was unable to differentiate nodes from a tumour on post-treatment scan. The second patient had a complete disappearance of the initial target lesion in a patient with multiple tumour lesions, requiring to use the hottest remaining lesion on post-treatment scan.

**Fig. 2:**
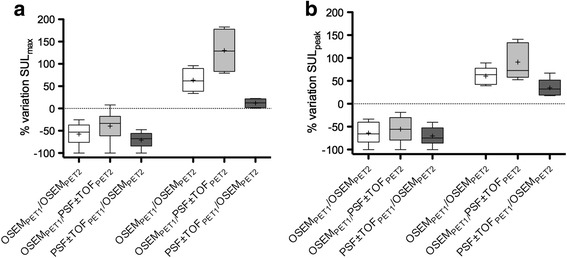
Impact of reconstruction consistency on the percentage variation in lesions SUL_max_ (**a**) and SUL_peak_ (**b**) in responding (*left panel*) and progressing (*right panel*) tumours. Data are shown as Tukey box plots. *Lines* denote median values as well as 10th and 90th percentiles. *Crosses* represent the mean values

The variations in SUL_max_ and SUL_peak_ between the pre- and post-treatment scans are shown in Fig. 2. For the OSEM_PET1_/OSEM_PET2_ scenario, which was taken as the reference standard, the change in SUL_max_ was −57.5% ± 23.4 and +63.4% ± 26.5 in the groups of tumours showing a decrease and an increase in ^18^F-FDG uptake, respectively. For SUL_peak_, it was −63.9% ± 22.4 and +60.7% ± 19.6, respectively.

The use of PSF reconstruction impacted SULs, depending whether this reconstruction was used for the pre- or post-treatment scans. For example, OSEM_PET1_/PSF ± TOF_PET2_ scenario reduced the apparent reduction in SUL in responding tumours (−39.7% ± 31.3 and −55.5% ± 26.3 for SUL_max_ and SUL_peak_, respectively) but increased the apparent increase in SUL in progressing tumours (+130.0% ± 50.7 and +91.1% ± 39.6 for SUL_max_ and SUL_peak_, respectively) as compared to the OSEM_PET1_/OSEM_PET2_ scenario described above. Accordingly, inconsistent reconstructions induced discordant response classifications amongst the different scenarios, as described in the section below.

### Impact of reconstruction-dependent variation of SUL on PERCIST and EORTC evaluation

**Fig. 3:**
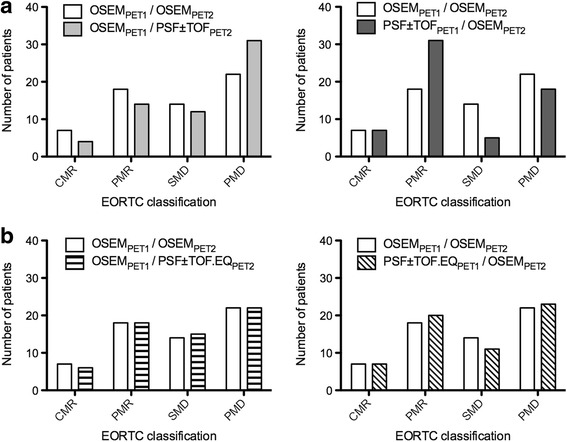
Impact of reconstruction inconsistency on EORTC classification. EORCT classification is shown for the standard of reference (OSEM_1_/OSEM_2_) and for other scenarios: reconstruction inconsistency between the baseline and post-treatment scans (**a**) and use of the EQ.PET methodology either for baseline or post-treatment scan (**b**)

**Fig. 4:**
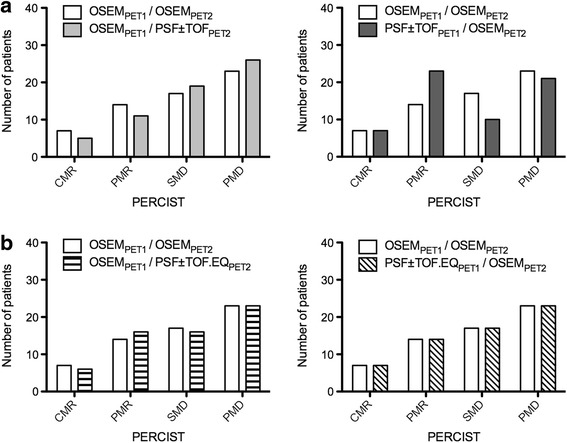
Impact of reconstruction inconsistency on PERCIST classification. PERCIST classification is shown for the standard of reference (OSEM_PET1_/OSEM_PET2_) and for other scenarios: reconstruction inconsistency between the baseline and post-treatment scans (**a**) and use of the EQ.PET methodology either for baseline or post-treatment scan (**b**)

By using OSEM for the pre- and post-treatment scans, PET classified 7 patients as CMR, 18 as PMR, 14 as SMD and 22 as PMD according to EORTC classification (Fig. 3) and 7 patients as CMR, 14 as PMR, 17 as SMD and 23 as PMD according to PERCIST (Fig. 4). According to EORTC evaluation, CMR occurred in five patients with a decrease in SUL_max_ to a level below the liver and blood pool background and in two patients to complete disappearance of the target lesions. PMD occurred in four patients with an increase in tumour SUL_max_ greater than 25% and in 18 patients with new lesions on the post-treatment scan. According to PERCIST classification, CMR occurred in five patients with a decrease in SUL_peak_ to a level below the liver and blood pool background and in two patients to complete disappearance of the target lesions. PMD occurred in five patients with an increase in tumour SUL_peak_ greater than 30% and in 18 patients with new lesions on the post-treatment scan.

The agreement level between EORTC and PERCIST therapeutic evaluations was almost perfect with a kappa value equal of 0.84 (0.73–0.95). Eight discordances (13%) occurred: one patient classified as CMR with EORTC and PMR with PERCIST, one patient classified as PMR with EORTC and CMR with PERCIST, four patients classified as PMR with EORTC and SMD with PERCIST and one patient classified as SMD with EORTC and PD with PERCIST.

Agreement levels between the OSEM_PET1_/OSEM_PET2_ scenario and other scenarios involving reconstruction inconsistency were found to be almost perfect with narrow confidence intervals for the scenarios using EQ.PET-filtered data either pre- or post-treatment and the reconstruction-consistent scenario for both EORCT and PERCIST classifications (Table 2). For EORTC and PERCIST evaluations, agreement levels were moderate to substantial for the scenario OSEM_PET1_/PSF ± TOF_PET2_ and PSF ± TOF_PET1_/OSEM_PET2_, with wide confidence intervals. Noticeably, kappa values were lower for EORTC classification than for PERCIST, especially for the OSEM_PET1_/PSF ± TOF_PET2_ scenario (0.55 quoted as moderate vs 0.77 quoted as substantial).

**Table 2:** Agreement levels between the OSEM_1_/OSEM_2_ scenario and other scenarios involving reconstruction inconsistency for EORTC and PERCIST therapeutic evaluations

	Kappa (95%CI)	
	EORTC	PERCIST
OSEM_PET1_/OSEM_PET2_ vs OSEM_PET1_/PSF ± TOF_PET2_	0.55 (0.39–0.72)	0.77 (0.63–0.90)
OSEM_PET1_/OSEM_PET2_ vs PSF ± TOF_PET1_/OSEM_PET2_	0.61 (0.45–0.77)	0.75 (0.62–0.88)
OSEM_PET1_/OSEM_PET2_ vs OSEM_PET1_/PSF ± TOF.EQ_PET2_	0.95 (0.89–1.00)	0.95 (0.89–1.00)
OSEM_PET1_/OSEM_PET2_ vs PSF ± TOF.EQ_PET1_/OSEM_PET2_	0.93 (0.86–1.00)	1.00 (1.00–1.00)
OSEM_PET1_/OSEM_PET2_ vs PSF ± TOF_PET1_/PSF ± TOF_PET2_	0.86 (0.75–0.97)	0.93 (0.85–1.00)
OSEM_PET1_/OSEM_PET2_ vs PSF ± TOF.EQ_PET1_/PSF ± TOF.EQ_PET2_	0.93 (0.85–1.00)	0.93 (0.85–1.00)

**Fig. 5:**
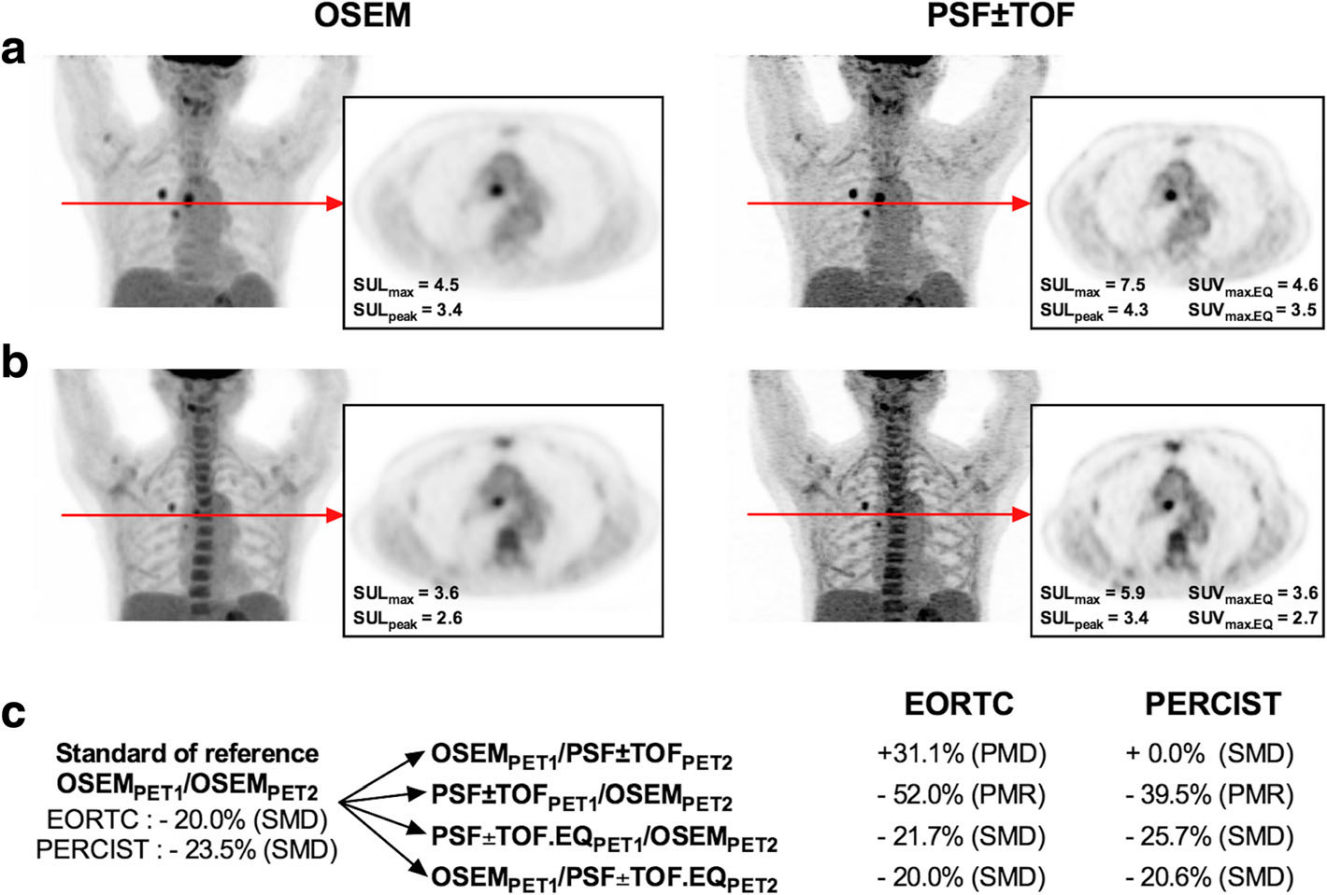
Representative images of a 66-year-old female with a NSCLC staged T1N2M0 or stage III according to AJCC stadification treated by chemotherapy. This patient was classified as SMD with EORTC classification and PERCIST according to the OSEM_PET1_/OSEM_PET2_ standard of reference, while OSEM_PET1_/PSF ± TOF_PET2_, a scenario mimicking a system upgrade during a trial led to a PMD with EORTC classification. The use of the EQ.PET methodology correctly classified the patient as SMD. **a** MIP images and transverse slices at the level of a mediastinal nodal involvement on OSEM and PSF ± TOF reconstructions for baseline scan. **b** MIP images and transverse slices at the level of a mediastinal nodal involvement on OSEM and PSF ± TOF reconstructions for post-treatment scans. **c** % change in SUL_max_ and SUL_peak_ for EORTC classification and PERCIST according to the different scenarios

Table 3 and Figs. 3 and 4 show the number of discordances in the EORTC and PERCIST classifications that occurred for the different scenarios tested. The EORTC classification displayed more discordances than what PERCIST did for all scenarios. For example, the scenario OSEM_PET1_/PSF ± TOF_PET2_ led to three patients being classified as PMR instead of CMR, seven as SMD instead of PMR, and nine as PMD instead of SMD with the EORTC classification whereas these same changes occurred, respectively, in two, five and three cases with the PERCIST classification. Figure 5 illustrates a patient classified as SMD according to the OSEM_PET1_/OSEM_PET2_ standard of reference with EORTC classification and PERCIST, while PSF + TOF_PET1/_OSEM_PET2_ led to PMR with both classifications and OSEM_PET1_/PSF + TOF_PET2_ led to PD with EORTC classification.

**Table 3:** Number of discordances between the OSEM_1_/OSEM_2_ scenario and other scenarios involving reconstruction inconsistency for EORTC and PERCIST therapeutic evaluations

	Numbers of discordances *n* (%)	
	EORTC	PERCIST
OSEM_PET1_/OSEM_PET2_ vs OSEM_PET1_/ PSF ± TOF_PET2_	19 (31)	10 (16)
OSEM_PET1_/OSEM_PET2_ vs PSF ± TOF_PET1_/OSEM_PET2_	17 (28)	11 (18)
OSEM_PET1_/OSEM_PET2_ vs OSEM_PET1_/ PSF ± TOF.EQ_PET2_	2 (3)	2 (3)
OSEM_PET1_/OSEM_PET2_ vs PSF ± TOF.EQ_PET1_/OSEM_PET2_	3 (5)	0 (0)
OSEM_PET1_/OSEM_PET2_ vs PSF ± TOF_PET1_/ PSF ± TOF_PET2_	6 (10)	3 (5)
OSEM_PET1_/OSEM_PET2_ vs PSF ± TOF.EQ_PET1_/ PSF ± TOF.EQ_PET2_	3 (5)	3 (5)

Consistent reconstruction (i.e. the PSF ± TOF_PET1_/PSF ± TOF_PET2_ and PSF ± TOF.EQ_PET1_/PSF ± TOF.EQ_PET2_ scenarios) did not give a perfect agreement compared to the OSEM_PET1_/OSEM_PET2_ standard of reference (Additional file 1: Figure S1). This was more pronounced for the EORTC classification in the PSF ± TOF_PET1_/PSF ± TOF_PET2_ scenario where six discordances occurred (Table 3), leading to a kappa value of 0.86 (Table 2).

## Discussion

In the framework of therapy monitoring with PET, pre- and post-treatment scans should ideally involve identical scan acquisition and image processing. However, this is often impractical in busy PET centres, especially those running several scanners. This can also be challenged by a scanner upgrade during the conduct of a trial or when a patient relocates. Previous studies aimed at validating the EARL harmonization strategy in the clinical setting have shown that SUV_max_ is more sensitive to reconstruction inconsistency than SUV_peak_ or their lean body mass equivalents, SUL_max_ and SUL_peak_. Consequently, one could expect a more significant impact of reconstruction inconsistencies on EORTC classification than on PERCIST.

In the present study, we evaluated the impact of inconsistent reconstruction on both EORTC and PERCIST response classifications, demonstrating variation in up to 31% of cases for EORTC classification vs up to 18% for PERCIST classification. Further, we showed that applying the EARL harmonization strategy provided more consistent response classification with kappa values greater than 0.93 for all the scenarios involving harmonized SULs, compared to the OSEM_PET1_/OSEM_PET2_ scenario used as a standard of reference. In line with its greater sensitivity to reconstruction inconsistencies, the EORTC classification benefited more from the EARL harmonization strategy, with kappa values increasing from 0.55 to 0.95 for the worst case scenario (OSEM_PET1_/PSF ± TOF_PET2_), compared with an improvement from 0.77 to 0.95 for PERCIST (Table 2).

This has practical advantages when there is variation of acquisition/reconstruction settings. This situation seems relatively common even in centres running the same PET system, as recently described by Sunderland and colleagues [25] in a survey involving 237 PET/CT systems in 170 international imaging centres with technology advancements spanning more than a decade, reporting that site-specific reconstruction parameters increased the quantitative variability of similar scanners, post-reconstruction smoothing filters being the most influential parameter. Harmonization has also practical advantages when the use of the same scanner for both scans is impractical, for instance in centres running two or more PET systems, as illustrated by the study by Skougaard et al. [26], in which 12 of 81 (14%) patients undergoing pre- and post-treatment PET in the same department were excluded for analysis because they were scanned on two different generation PET systems.

Taking, for example, the scenario of a system upgrade during a trial, the use of OSEM for the pre-treatment scan while using PSF ± TOF for the post-treatment scan led to discordant response assessments in 19/61 (31%) for EORTC classification and 10/61 (16%) for PERCIST (Table 3). Using a harmonization strategy (hereby aligning quantitative values to the EARL/EANM harmonizing standards with a proprietary filter, the EQ.PET methodology) either for the pre- or post-treatment scans gave almost perfect agreement levels in comparison with the OSEM_PET1_/OSEM_PET2_ reference standard, with narrow confidence intervals. We observed only two discordances for the OSEM_PET1_/PSF ± TOF.EQ_PET2_ vs OSEM_PET1_/OSEM_PET2_ scenario for both the EORTC and PERCIST classifications and three discordances which occurred for the PSF ± TOF.EQ_PET1_/OSEM_PET2_ vs OSEM_PET1_/OSEM_PET2_ scenario for the EORTC classification. No discordance occurred for the PSF ± TOF.EQ_PET1_/OSEM_PET2_ vs OSEM_PET1_/OSEM_PET2_ scenario for PERCIST classification. The three discordances that occurred only with EORTC classification for the PSF ± TOF.EQ_PET1_/OSEM_PET2_ were due to SUL_max_ variations between the pre and post-treatment scans very close to the cut-off value of +25 or −25% with the standard scenario OSEM_PET1_/OSEM_PET2_ resulting in changes from SMD to either PMR or PMD and vice versa for other scenarios.

It is noteworthy that consistent reconstruction (i.e. the PSF ± TOF_PET1_/PSF ± TOF_PET2_ and PSF ± TOF.EQ_PET1_/ PSF ± TOF.EQ_PET2_ scenarios) did not give perfect agreement compared to the OSEM_PET1_/OSEM_PET2_ standard of reference. These discordances were due to PSF reconstruction increasing SUV metrics in the tumours while not impacting the background (blood pool and liver) [27, 28], leading to CMR being changed to PMR. Also, both the EORTC and PERCIST classifications were affected by %change in SUL close to +30%/+25% or −30%/−25% for the OSEM_PET1_/OSEM_PET2_ scenario resulting in changes from SMD to either PMR or PMD and vice versa for other scenarios.

A limitation of this study is that we used EQ.PET, a software solution developed for and applied only to scanners and reconstruction algorithms of the company that developed this product. EQ.PET has not been validated for equipment from other manufacturers but has been shown to be as effective as the alternative approach of obtaining a second reconstruction dataset, as recommended by the EARL accreditation program for quantitation [29, 30]. The ability of this algorithm to correct for scans performed on different scanners and then processed with different reconstruction methods was not tested.

## Conclusions

PERCIST classification is less sensitive to reconstruction algorithm-dependent variability than EORTC classification. The EORTC and PERCIST classifications would benefit from harmonization strategies such as the EARL accreditation program in multicentre studies or in sites equipped with multiple PET systems.

## Additional file

Additional file 1: Figure S1. Impact of reconstruction consistency on EORTC classification and PERCIST. EORCT classification and PERCIST are shown for the standard of reference (OSEM_1_/OSEM_2_) and for other scenarios involving reconstruction consistency between the baseline and post-treatment scans using either PSF ± TOF (a) or the EQPET methodology (PSF ± TOF.EQ; b). (TIFF 1787 kb)
