# Supplementary figures and images for: EORTC PET response criteria are more influenced by reconstruction inconsistencies than PERCIST but both benefit from the EARL harmonization program
Source: EJNMMI Phys. 2017 May 30;4:17. doi: 10.1186/s40658-017-0185-4 (PMC5449363; doi:10.1186/s40658-017-0185-4)

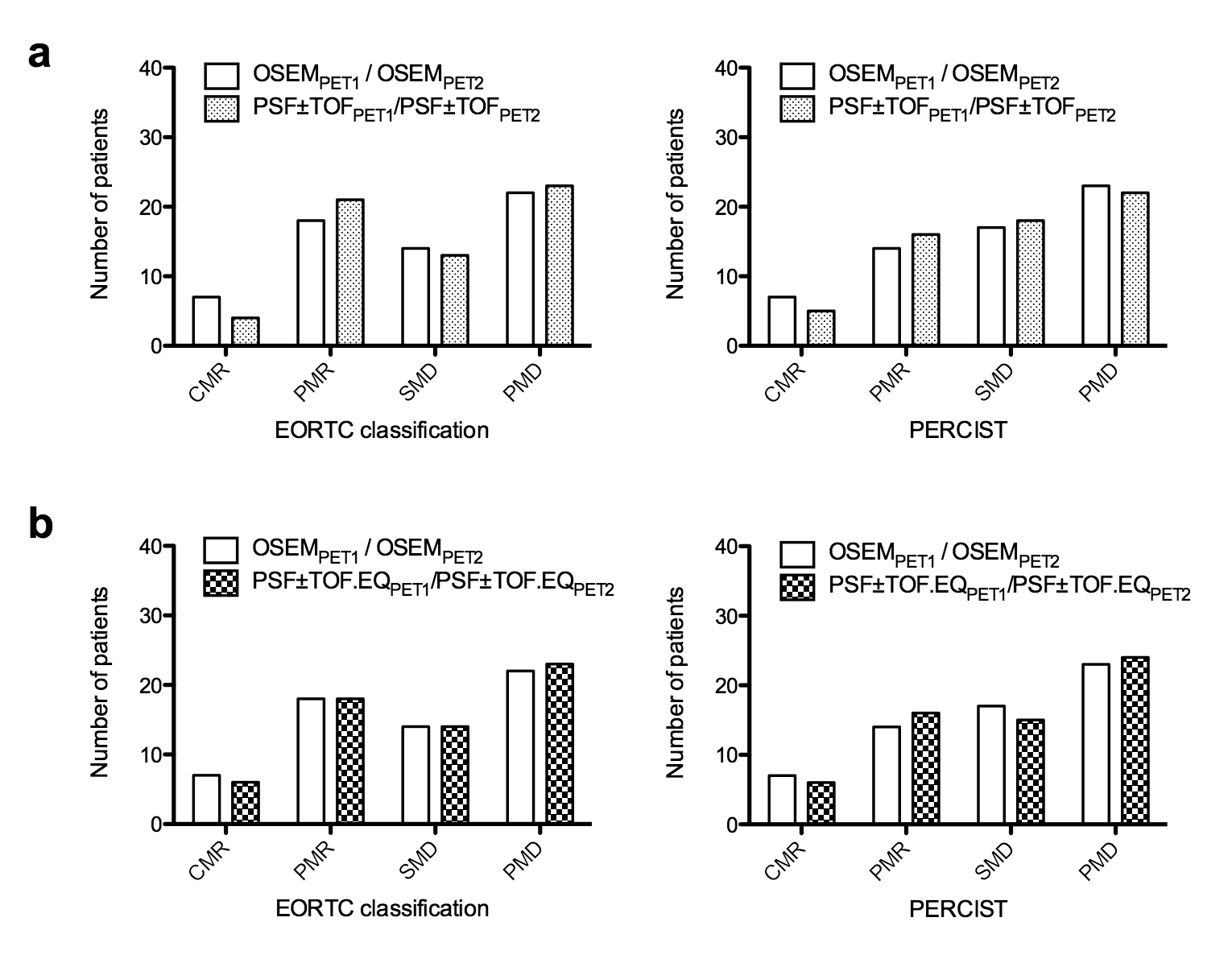

Supplement: Additional file 1: Figure S1. — Impact of reconstruction consistency on EORTC classification and PERCIST. EORCT classification and PERCIST are shown for the standard of reference (OSEM1/OSEM2) and for other scenarios involving reconstruction consistency between the baseline and post-treatment scans using either PSF ± TOF (a) or the EQPET methodology (PSF ± TOF.EQ; b). (TIFF 1787 kb) [file 40658_2017_185_MOESM1_ESM.tiff]
